# Caste- and age-specific venom composition of biogenic amines and the influence of diet in honey bees

**DOI:** 10.1371/journal.pone.0338795

**Published:** 2025-12-10

**Authors:** Motoharu Hamano, Kazuya Niki, Keiki Imamura, Ken Sasaki

**Affiliations:** 1 Graduate School of Agriculture, Tamagawa University, Machida, Tokyo, Japan; 2 Department of Agri-Production Sciences, Tamagawa University, Machida, Tokyo, Japan; University of Leipzig Faculty of Life Sciences: Universitat Leipzig Fakultat fur Lebenswissenschaften, GERMANY

## Abstract

The chemical composition of the venom in social Hymenoptera differs between castes and ages. Biogenic amines are contained in the venom of honey bees and may be physiologically effective to vertebrate predators and insects. This study quantified the concentrations of biogenic amines in venom and compared them between different castes and ages of honey bees. The concentrations of dopamine and *N*-acetyldopamine in venom were significantly higher in virgin queens than workers of the same age. The concentrations of dopamine, norepinephrine, tyramine and serotonin increased with age in virgin queens and workers. There was a significant positive correlation between venom dopamine concentrations and ovarian development in queenless workers, suggesting that the concentration of dopamine in the venom transformed from normal workers to reproductive females as that in virgin queens. We also tested the possibility of dietary effects on the concentration of dopamine in venom. Workers fed tyrosine or royal jelly showed significantly higher concentrations of dopamine precursors, tyrosine, 3,4-dihydroxyphenylalanine (DOPA) and dopamine in the hemolymph, as well as higher concentrations of dopamine in venom than in controlled workers. These results suggest that compositions of biogenic amines in venom are influenced by nutrition and change based on their social roles in honey bee society.

## Introduction

The chemical compositions of venom in social Hymenoptera have evolved to defend the nest from predators [1,2]. The sting targets of workers are insects and vertebrate predators including mammals. Therefore, the chemical compositions of venom in workers are effective to not only the insect’s physiological activities [1–3] but also the vertebrate physiological and immune responses [4,5]. In workers of several eusocial species, the sting apparatus with venom sac, as well as its motor system, can be separated from the abdomen during stinging; a process called stinging autotomy, which facilitates the sting movement and venom injection [6–9]. Such sting autotomy does not occur in queens [7,8]. Thus, the stinging system is specialized in each caste.

Venom in the honey bee *Apis mellifera* contains peptides, biogenic amines, phospholipids, amino acids, carbohydrates and mineral substances [1,4,5,10–12]. The concentration of each component in venom differs between castes and ages [13,14]. It has also been shown that the concentration of each component of the venom varies depending on the season [15] and nest location [16], which may be associated with sources of nectar or pollen. However, such factors remain to be tested experimentally.

Biogenic amines are neuroactive substances that modulate neural activities and control diverse behaviors in vertebrates and invertebrates [2,17–21]. The presence of biogenic amines in the venom of the honey bees has been reported [10,11,22,23]. The venom in workers functions as a defensive weapon for nests, whereas the queen’s venom is used during battle between sister virgin queens [8,24]. The differences in the concentrations of biogenic amines between castes or ages have been implicated [11,23,25,26], however, the physiological mechanisms underlying the regulation of concentrations of venom components remain unclear.

The present study investigated the concentrations of biogenic amines in the venom in queens, workers, and queenless reproductive females of honey bees. Since those females may have different nutritional states in the colony, we hypothesized that nutrition can affect the concentrations of biogenic amines in venom. Therefore, we tested the hypothesis by manipulating diets under experimental conditions. Those experiments revealed that the intake of a tyrosine-rich diet affects dopamine concentrations in venom.

## Materials and methods

### Honey bee colonies

To obtain workers of the honey bee *A. mellifera*, the frames containing worker pupae were removed from the hives a few days before emergence and transferred to an incubator heated at 34°C. Newly emerged workers (0 days old) were marked with paint on the thorax to identify their age. Marked bees were then returned to the mother colonies. At the same time, several 0-day-old individuals were collected for their venom. Workers introduced into the mother colonies were then collected at specific day-ages (5, 10, and 15 days).

To obtain queen bees, queens were reared using a standard queen-rearing method with queenright colonies [27]. Briefly, newly hatched larvae in worker comb cells from queenright colonies were placed into plastic queen cups and returned to the colonies with two-storied hives. The larvae grafted into queen cups were placed in a queenless area created using a horizontal queen excluder in the queenright colonies. The sealed queen cells were then removed from the colonies and transferred to an incubator 2 days before emergence. They were held in individual plastic containers (60 mm height × 45 mm i.d.) at 34˚C until harvested as newly emerged adults. The emerged queens were individually held in a small cage (85 mm length × 40 mm width × 20 mm height) with 10 nurse bees until 5 days of age. At the same time, several 0-day-old individuals were collected for their venom.

### Collection of venom and hemolymph

The head of workers or queens was frozen and immobilized using a cold spray gun (Spot freeze, Fine Chemical Japan, Osaka, Japan). The sting chamber with a venom sac was then removed from the abdomen. The venom was collected from the venom sac using a glass capillary with scales (Microcaps, Drummond Scientific, PA, USA). The collected venom was then mixed with ice-cold 0.1M perchloric acid containing 0.1 ng/μL 3,4-dihydroxybenzylamine as an internal standard. Each sample was then centrifuged at 15,000 *g* for 15 min at 4˚C. Supernatants were transferred to microvials for analysis by high-performance liquid chromatography with electrochemical detection (HPLC-ECD). Hemolymph was collected from the abdomen using a glass capillary and treated using the same procedure as for the venom samples.

### Measurements of biogenic amines

Two HPLC-ECD systems were used to measure the concentrations of biogenic amines in venom and hemolymph. Dopamine, tyramine, *N*-acetyldopamine (dopamine metabolite), and serotonin were quantified using the analytical system A, whereas tyrosine, 3,4-dihydroxyphenylalanine (DOPA) (dopamine precursors), and norepinephrine (dopamine metabolite) were quantified using another system (analytical system B) [28].

Analytical system A comprised a solvent delivery pump (PU-4180, JASCO, Tokyo, Japan), refrigerated automatic injector (AS-4050, JASCO), and C18 reversed-phase column (250 mm × 4.6 mm id., 5-μm average particle size, UG120, Osaka Soda, Osaka, Japan) maintained at 35°C. An electrochemical detector (ECD-700, EICOM, Kyoto, Japan) was set at 0.8 V and held at 35°C. The mobile phase contained 0.18 M monochloroacetic acid and 40 μM 2Na-EDTA, which was adjusted to pH 3.6 with NaOH; 1.62 mM sodium-1-octanesulfonate and 5% CH_3_CN were then added. The flow rate was kept constant at 0.7 mL/min.

Analytical system B comprised a solvent delivery pump (PU-4580, JASCO), refrigerated automatic injector (AS-4550, JASCO), and C18 reversed-phase column (250 mm × 4.6 mm id., 5-μm average particle size, MG, Osaka Soda) maintained at 35°C. An electrochemical detector (ECD-700, EICOM) was set at 0.87 V under 35°C. The mobile phase contained 83 mM citric acid monohydrate, 17 mM sodium acetate, 13 μM 2Na-EDTA, 2.3 mM sodium-1-octanesulfonate, and 7% methanol. The flow rate was held constant at 0.7 mL/min.

In both HPLC-ECD systems, external standards were run before and after the sample runs to identify and quantify the dopamine-related substances (tyrosine, DOPA, dopamine, norepinephrine and *N*-acetyldopamine), tyramine and serotonin. Peaks of those substances were identified by comparing the retention times and hydrodynamic voltammograms to those of standards. Measurements based on the peak area of the chromatogram were determined by calculating the ratio of the peak area of the substance to the peak area of the standard.

### Evaluation of ovary development in reproductive workers

Workers can develop ovaries under queenless conditions. Newly emerged workers were marked on the thorax and then introduced into queenless colonies. After 15 days, marked females were collected from the colonies. The venom was collected from the venom sac using a glass capillary with scales for measurements of biogenic amines. Ovary activity was evaluated by measuring the length of the largest terminal (basal) oocytes in the ovarioles. Each pair of ovaries was carefully removed from the abdomen under a dissecting microscope. Photographic images were taken with a digital camera and analyzed with commercially available computer software (Photomeasure, Kenis, Osaka, Japan).

### Oral application of tyrosine and royal jelly

Newly emerged workers were marked with paint on the thorax to identify their age. Marked bees were then returned to the mother colonies. After 4 days, 20–25 marked workers (4 days old) with 40–50 nurse workers (unknown age) were collected from the mother colony and were held in a wooden box (11.7 cm × 11.7 cm × 6.2 cm) covered with a steel net and a floor coated in beeswax. For tyrosine oral application, each group of 20–25 workers was provided *ad libitum* access to either 1.0 mg/ml tyrosine (Tyr) in 30% sucrose solution or 30% sucrose solution (control), and pollen cakes in an incubator at 30°C for 4 or 8 days. For the feeding of royal jelly, each group of 20–25 marked workers (4 days old) was provided *ad libitum* access to a 1:1 mixture of royal jelly (Roy) and 30% sucrose solution or 30% sucrose solution (control), and pollen cakes for 4 or 8 days. Royal jelly was collected by a commercial procedure using plastic queen cells and stored at 4 °C until use. After 4 or 8 days of feeding, the marked workers (8 or 12 days old) were immobilized using a cold spray gun, and hemolymph or venom was collected using the procedure described above and analyzed by HPLC-ECD. Workers from three colonies were used in the experiments.

### Statistics

The data did not meet the criteria for parametric tests, and therefore, nonparametric tests were used for the analyses. The concentrations of biogenic amines between castes were examined by the Mann-Whitney U test (significance level = 0.05). The concentrations between workers and queens of different ages were examined by the Kruskal-Wallis test (significance level = 0.05) with the Steel-Dwass multiple comparison test (significance level = 0.05, this level was adjusted to reflect the number of the tests) or the Mann-Whitney U test, respectively. In reproductive workers, the correlation between amine concentrations and maximum oocyte lengths were tested using Spearman’s rank correlation. In tyrosine-fed or royal jelly-fed experiments, the concentrations of biogenic amines between treated and control workers were compared by the Mann-Whitney U test.

## Results

### Biogenic amine concentrations in venom between castes at different ages

**Fig 1:**
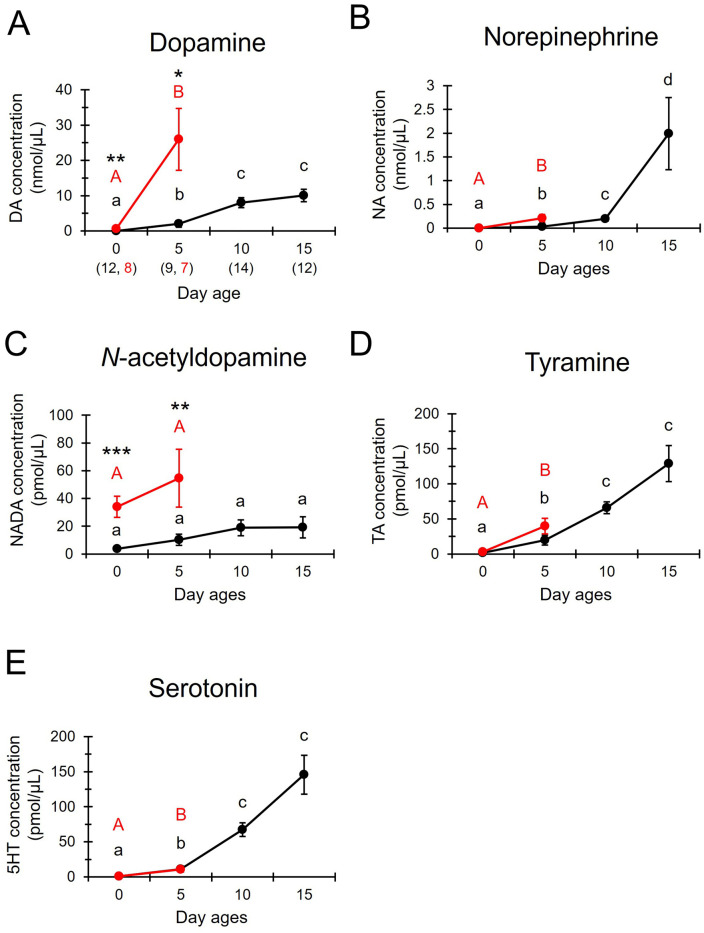
Biogenic amine concentrations in venom from virgin queens and workers at different ages. A: concentration of dopamine (DA), B: norepinephrine (NA), C: *N*-acetyldopamine (NADA), D: tyramine (TA), E: serotonin (5HT). The concentrations (mean ± standard error) in 0- and 5-day-old queens (red, N = 8 and 7, respectively) and 0-, 5-, 10- and 15-day-old workers (black, N = 12, 9, 14 and 12, respectively) are indicated. The values in parentheses in Fig 1A indicate the numbers of individuals examined (left: worker, right: queen) and are applicable to all panels **(A-E)**. Differences between ages within the same caste examined by the Mann-Whitney U test (between queens, significance level = 0.05) or the Kruskal-Wallis test (among workers, significance level = 0.05) with the Steel-Dwass multiple comparison test (adjusted significance level = 0.05) are indicated by different letters (uppercase letter: queen, lowercase letter: worker). Caste differences tested by the Mann-Whitney U test are shown with an asterisk (*: P < 0.05, **: P < 0.01, ***: P < 0.001).

Biogenic amines including dopamine, norepinephrine, *N*-acetyldopamine, tyramine, and serotonin were detected in the venom of virgin queens and workers (Fig 1). The concentrations of dopamine and norepinephrine were particularly high compared to others in the nanomole range (Fig 1A and 1B). In workers, the concentrations of dopamine, norepinephrine, tyramine, and serotonin in venom were significantly higher in 10-day-old individuals than in 0-day-old or 5-day-old individuals (Kruskal-Wallis test, dopamine: H = 31.492, P < 0.0001, Fig 1A; norepinephrine: H = 35.778, P < 0.0001, Fig 1B; tyramine: H = 33.467, P < 0.0001, Fig 1D; serotonin: H = 36.353, P < 0.0001, Fig 1E, Steel-Dwass, P < 0.05), whereas those of *N*-acetyldopamine did not differ between ages (Kruskal-Wallis test, H = 7.675, P = 0.053, Fig 1C). In virgin queens, the concentrations of dopamine, norepinephrine, tyramine, and serotonin in venom were significantly higher in 5-day-old individuals than 0-day-old individuals (Mann-Whitney U test, dopamine: z = 2.315, P < 0.05, Fig 1A; norepinephrine: z = 3.240, P < 0.01, Fig 1B; tyramine: z = 2.546, P < 0.05, Fig 1D; serotonin: z = 1.967, P < 0.05, Fig 1E), whereas those of *N*-acetyldopamine did not differ between ages (U test, z = 0.579, P = 0.563, Fig 1C).

Caste differences in the concentrations of dopamine and *N*-acetyldopamine were detected at 0 and 5 days of age (Fig 1A and 1C). The concentrations of dopamine and *N*-acetyldopamine were significantly higher in 0- and 5-day-old queens than workers of the same age (Mann-Whitney U test, dopamine: 0 days: z = 2.777, P < 0.01; 5 days: z = 2.170, P < 0.05, Fig 1A; *N*-acetyldopamine: 0 days: z = 3.472, P < 0.001; 5 days: z = 2.699, P < 0.01, Fig 1C), whereas the concentrations of norepinephrine, tyramine, and serotonin were not significantly different between castes (U test, norepinephrine: 0 days: z = 1.157, P = 0.247; 5 days: z = 1.747, P = 0.081, Fig 1B; tyramine: 0 days: z = 1.466, P = 0.143; 5 days: z = 1.852, P = 0.064, Fig 1D; serotonin: 0 days: z = 0.772, P = 0.44; 5 days: z = 0.159, P = 0.874, Fig 1E).

### Biogenic amine concentrations in venom of reproductive workers

**Fig 2:**
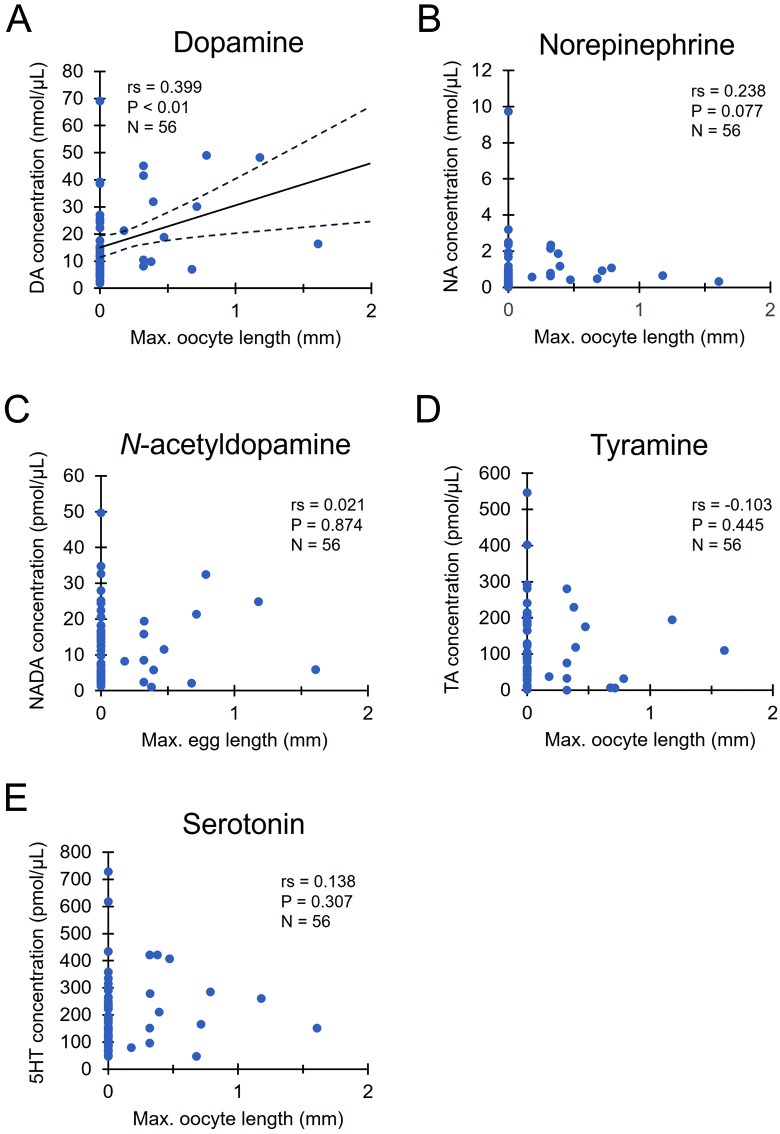
Correlation between biogenic amine concentrations in venom and the maximum oocyte lengths in queenless workers. Correlations were determined based on Spearman’s correlation coefficients (rs). Dotted lines in Fig 2A represent the 95% confidence interval.

Under queenless conditions, several workers had well-developed ovaries and were potential egg-laying workers. Ovary development was evaluated based on the maximum oocyte length. The concentrations of dopamine in venom were positively correlated to the maximum oocyte lengths in the ovarioles (Spearman’s rank correlation, rs = 0.399, P < 0.01, Fig 2A). However, the concentrations of norepinephrine, *N*-acetyldopamine, tyramine, and serotonin were not significantly correlated with the maximum oocyte lengths (norepinephrine: rs = 0.238, P = 0.077, Fig 2B; *N*-acetyldopamine: rs = 0.021, P = 0.874, Fig 2C; tyramine: rs = −0.103, P = 0.445, Fig 2D; serotonin: rs = 0.138, P = 0.307, Fig 2E).

### Effects of tyrosine intake on the concentrations of biogenic amines in venom

**Fig 3:**
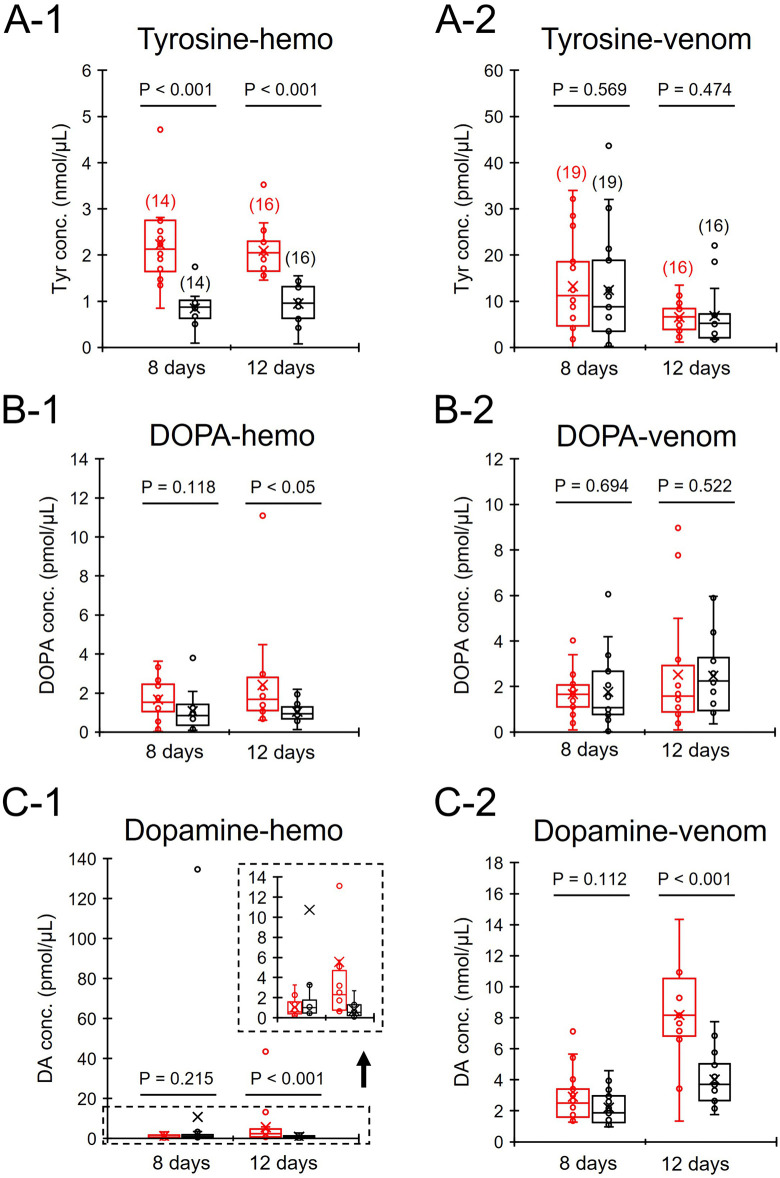
Concentrations of dopamine-related substances in the hemolymph and venom in tyrosine-fed (red) and control workers (black). The values in parentheses in Fig 3A-1 and 3A-2 indicate the number of individuals examined and are applicable to all panels **(A-C)**. In Fig 3C-1, the dashed area is a magnified inset graph. Box and whisker plots display the median (the line inside the box), the first and third quartiles (the upper and lower outlines of the box), the minimum and maximum values (the whiskers) and the mean (the crosses). P values examined by the Mann-Whitney U test (significance level = 0.05) are indicated.

Workers orally treated with tyrosine at 8 and 12 days of age had significantly higher concentrations of tyrosine in hemolymph than control workers (Mann-Whitney U test, 8 days old: z = 4.0, P < 0.001; 12 days old: z = 4.749, P < 0.001, Fig 3A-1). They also had significantly higher concentrations of DOPA and dopamine at 12 days of age in hemolymph than control workers (U test, DOPA: z = 2.563, P < 0.05, Fig 3B-1; dopamine: z = 3.392, P < 0.001, Fig 3C-1). However, the concentrations of DOPA and dopamine in hemolymph at 8 days of age did not differ between Tyr-fed and control workers (U test, DOPA: z = 1.562, P = 0.118; dopamine: z = 1.241, P = 0.215).

The concentrations of tyrosine and DOPA in venom did not differ between Tyr-fed and control workers at 8 and 12 days of age (U test, tyrosine 8 days old: z = 0.569, P = 0.569; tyrosine 12 days old: z = 0.716, P = 0.474, Fig 3A-2; DOPA 8 days old: z = 0.394, P = 0.694; DOPA 12 days old: z = 0.641, P = 0.522, Fig 3B-2). The concentrations of dopamine in venom at 12 days of age were significantly higher in Tyr-fed workers than control workers (U test, z = 3.58, P < 0.001, Fig 3C-2), whereas the concentrations of dopamine at 8 days of age did not differ between Tyr-fed and control workers (U test, z = 1.591, P = 0.112).

### Effects of royal jelly intake on the concentrations of biogenic amines in venom

**Fig 4:**
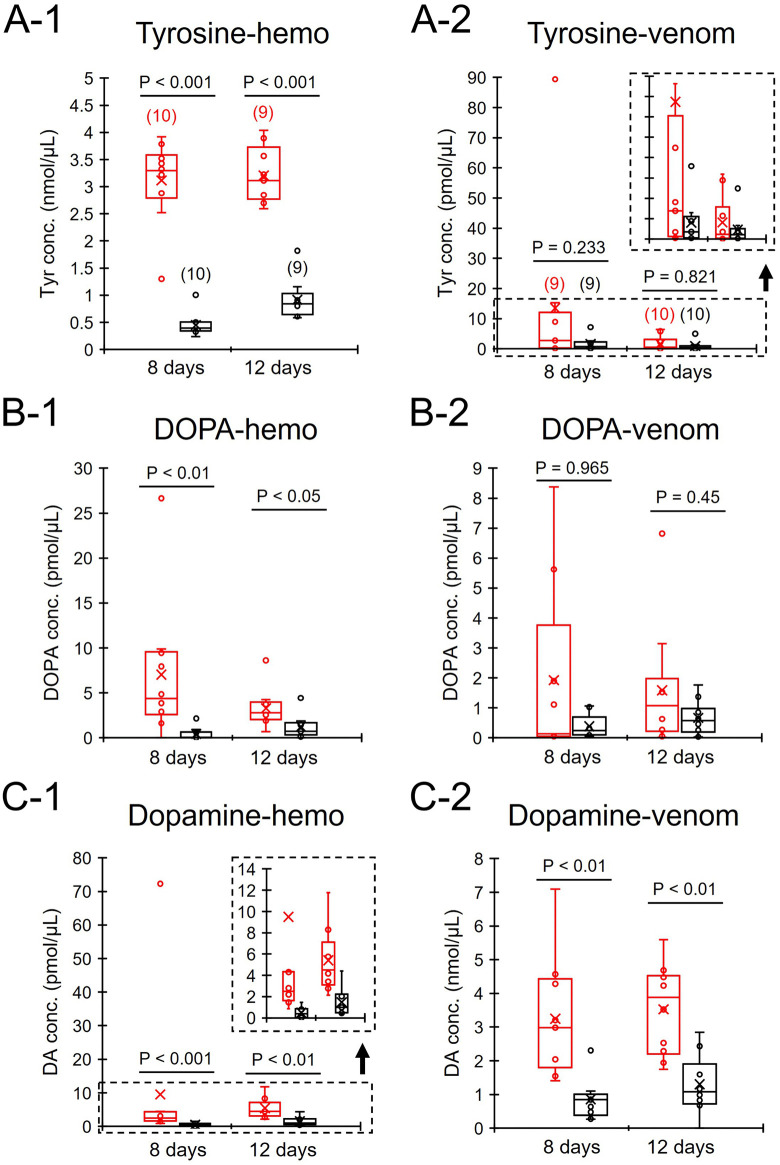
Concentrations of dopamine-related substances in the hemolymph and venom in royal jelly-fed (red) and control workers (black). The values in parentheses in Fig 4A-1 and 4A-2 indicate the number of individuals examined and are applicable to all panels **(A-C)**. In Figs A-2 and C-1, the dashed areas are magnified inset graphs. Box and whisker plots display the median (the line inside the box), the first and third quartiles (the upper and lower outlines of the box), the minimum and maximum values (the whiskers) and the mean (the crosses). P values examined by the Mann-Whitney U test (significance level = 0.05) are indicated.

Workers fed royal jelly for 8 and 12 days had significantly higher concentrations of tyrosine, DOPA, and dopamine in hemolymph than control workers (Mann-Whitney U test, tyrosine 8 days old: z = 3.78, P < 0.001; tyrosine 12 days old: z = 3.576, p < 0.001, Fig 4A-1; DOPA 8 days old: z = 2.948, P < 0.01; DOPA 12 days old: z = 2.517, P < 0.05, Fig 4B-1; dopamine 8 days old: z = 3.553, P < 0.001; dopamine 12 days old: z = 3.135, P < 0.01, Fig 4C-1).

The concentrations of tyrosine and DOPA in venom did not differ between Roy-fed and control workers (U test, tyrosine 8 days old: z = 1.192, P = 0.233; tyrosine 12 days old: z = 0.227, P = 0.821, Fig 4A-2; DOPA 8 days old: z = 0.044, P = 0.965; DOPA 12 days old: z = 0.756, P = 0.45, Fig 4B-2). However, the concentrations of dopamine were significantly higher in workers fed royal jelly at 8 days and 12 days of age than in control workers of the same age (U test, 8 days old: z = 3.223, P < 0.01; 12 days old: z = 3.25, P < 0.01, Fig 4C-2).

## Discussion

Honey bee venom contains various bioactive substances, and the concentrations of the components differ between seasons [11,15] and nest sites [16]. Biogenic amines are important components of venom causing increased heartrates, pain, and inflammation to mammals, including humans [2,4,5]. Different concentrations of biogenic amines between castes or worker ages have been reported [11,23,26], however, the physiological mechanisms regulating the concentrations of each component in venom remain unclear. This study revealed the caste- and age-specific concentrations of biogenic amines in venom and determined correlations between dopamine concentrations and ovarian development in reproductive workers. We also demonstrated that food containing the dopamine precursor, tyrosine, could enhance dopamine concentrations in venom, suggesting that food can alter the concentrations of biogenic amines in the venom of honey bees via the hemolymph.

### Caste-specific concentrations of dopamine in venom

Comparisons of the concentrations of biogenic amines in the venom between virgin queens and workers showed that dopamine concentrations were significantly higher in 0- and 5-day-old queens than the same aged workers. Virgin queens use venom to attack rival sister queens when they encounter one another in the colony [8,24]. Since they need to produce effective venom against adult honey bees as soon as possible following emergence, the compositions of biogenic amines in venom, especially dopamine, may be immediately synthesized after the emergence of queens. A similar early synthesis of venom components (melittine and promelittine) in virgin queens has also been reported [13]. However, it is unknown whether dopamine is more effective against insects than vertebrates.

Dopamine concentrations in venom were positively correlated with the maximum oocyte lengths in ovarioles. However, the significant positive correlation was not detected for tyramine, norepinephrine, *N*-acetyldopamine, or serotonin. Workers with developed ovaries are potential reproductive workers and may maintain the physiological states for laying eggs. It has been reported that reproductive workers have high levels of aggressiveness against nest mates and occasionally expose the stinger [29], although it is unclear whether they use the venom during such aggressive behaviors. The high concentrations of dopamine in the venom of queenless workers with developed ovaries are similar to those of virgin queens. The dopamine in the venom may be an effective component for reproductive females under competition between rival females.

The biological function of venom dopamine remains to be tested. In honey bees, a single injection of similar concentrations of dopamine in venom causes neuromodulatory effects on the behavior but no toxicity [30–32]. Rather, dopamine may help distribute other toxic components of the venom throughout the target’s body through its neuromodulatory effects. Since virgin queens use venom to compete with other queens, dopamine may act effectively on the insect body along with other components of the venom.

Queen-specific high concentrations of dopamine in the brains and hemolymph have been reported in honey bees [33,34]. Queenless workers with developed ovaries also have high dopamine concentrations in the brains [35,36]. Dopamine levels in the brain of queenless workers are influenced by tyrosine levels in food [37]. Although the function of dopamine may differ between the brain and venom, the regulation of dopamine synthesis in both tissues may be influenced by dietary tyrosine. Since the nutritional states in queens are similar to that of reproductive workers, but differ from normal workers, nutrition may be important to understand the regulation of dopamine synthesis in venom.

### Age-specific concentrations of biogenic amines in venom

Age-related differences in the concentrations of dopamine, norepinephrine, tyramine, and serotonin were detected in workers from the time of emergence to day 15. Guard workers usually require about two weeks after emergence for age-polyethism [8]. Therefore, it is possible that the components of the venom are already complete before the guard bees are ready to use it. Because the concentrations of several biogenic amines we measured increased by day 15, these amines may be necessary components of the venom to be effective against vertebrate predators, particularly mammals.

In venom, high concentrations of dopamine and norepinephrine were detected. Those results suggested that dopamine was metabolized into norepinephrine in venom. In contrast, dopamine in the brains was primarily converted into *N*-acetyldopamine and to a lesser extent into norepinephrine [33,36]. The differing dopamine metabolisms between brains and venom may be due to the function of norepinephrine as a neurotransmitter that can be effective to the nervous and muscular systems of mammals. When venom containing norepinephrine is injected into mammals, norepinephrine increases the heartrate or pulse, pain, or inflammation [4,5].

### Effects of food intake on the composition of biogenic amines in venom

It has been reported that the concentrations of dopamine in venom differ between castes and seasons [23]. This study identified different concentrations of dopamine between reproductive states among workers. The diverse dopamine concentrations in venom can be explained by the intake of different levels of tyrosine in food. Our tyrosine-feed experiments showed that workers fed tyrosine had high concentrations of tyrosine at 8 and 12 days of age, and high concentrations of DOPA and dopamine at 12 days of age in hemolymph. Those data suggest that the tyrosine in food was absorbed from the gut and moved into the hemolymph. Similarly, DOPA and dopamine may accumulate in hemolymph until 12 days of age. However, in venom, only the dopamine concentrations were significantly higher in tyrosine-fed workers than in control workers. Those results suggest that the tyrosine and DOPA in hemolymph may be used as the material for synthesizing dopamine in venom glands, but may not be stored in venom sac. Alternatively, it is possible that only dopamine in the hemolymph was transported to the venom sac. The no dietary effect on the levels of tyrosine and DOPA in venom was the case with the royal jelly-fed workers. Tyrosine intake from royal jelly feeding was more effective than that from tyrosine feeding, however, the concentrations of tyrosine and DOPA were not different in venom between royal jelly-fed and control workers. The concentrations of dopamine at 8 and 12 days of age were significantly higher in royal jelly-fed workers than in control workers. Thus, nutrients from food potentially influence the concentrations of venom components, which may generate diverse compositions of honey bee venoms depending on season, nest site, and reproductive states of workers.

The intake of tyrosine-rich diet may not only provide precursors of dopamine as substrates but also contribute to the activation of the expression of enzyme genes involved in dopamine synthesis. It has been reported that tyrosine intakes stimulate gene expression of tyrosine hydroxylase and DOPA decarboxylase in the brain, increasing dopamine levels in the brain of male honey bees [38]. A similar activation of the gene expression involved in dopamine synthesis may occur in the venom gland. Further, royal jelly contains many potential bioactive components [39] and may have additional effects on dopamine synthesis in the venom gland. The results of the effective increase in venom dopamine by royal jelly feeding may be due to the additional effects.

From a perspective of behavioral ecology, food supply changes seasonally, affecting the production of reproductive individuals in the colony. Under nutrient-rich conditions, colonies can produce more nutritious queens with effective venom containing more dopamine. This relationship between nutritional state and effective venom leads to the selection of superior queens through competition between virgin queens. In workers, the high concentrations of dopamine in venom may be converted to high concentration of norepinephrine, which may act more effectively on vertebrate physiology. Since colonies have more brood and food stores under nutrient-rich conditions, workers may be able to defend more valuable colonies with more effective venom.

## Conclusion

This study investigated caste- and age-specific concentrations of biogenic amines and their dietary influence in the venom of honey bees. High concentrations of dopamine in venom were queen specific. The concentrations of dopamine, norepinephrine, tyramine, and serotonin increased with age in workers and virgin queens. Positive correlations were observed between dopamine concentrations in venom and ovarian development among queenless workers, thus, suggesting that a queen-like composition of venom in reproductive workers. The diverse compositions of venom in honey bees can be explained by the intake of different amounts of tyrosine from food. This study showed that tyrosine-fed or royal jelly-fed workers had higher concentrations of tyrosine, DOPA, and dopamine in hemolymph, but higher concentrations of only dopamine in venom. Those results suggest that tyrosine intake from food influences the concentrations of dopamine in venom.

## Supporting information

S1 Table Data of concentrations of biogenic amines in the venom of workers and virgin queens. (PDF)

S2 Table Data of concentrations of biogenic amines in the venom and maximum oocyte lengths in queenless workers. (PDF)

S3 Table Data of concentrations of dopamine-related substances in the hemolymph and venom in tyrosine-fed and control workers. (PDF)

S4 Table Data of concentrations of dopamine-related substances in the hemolymph and venom in royal jelly-fed and control workers. (PDF)

S5 Table P values of the Steel-Dwass multiple comparison test between different ages in workers (Fig 1A–1E). (PDF)
