# Supplementary material for: Caste- and age-specific venom composition of biogenic amines and the influence of diet in honey bees
Source: PLoS One. 2025 Dec 10;20(12):e0338795. doi: 10.1371/journal.pone.0338795 (PMC12694850; doi:10.1371/journal.pone.0338795)
Supplement: S1 Table — (PDF) [file pone.0338795.s001.pdf]

S1 Table. Data of concentrations of biogenic amines in the venom of workers and virgin queens.

Dopamine (pmol / µL)

Worker				Queen	
0	4-5	9-10	15	0	5
17.63274	9.4254	3496.916	2682.631	20.96153	24043.51
136.1925	211.3917	1437.98	1928.965	1167.494	15844.05
62.56508	2127.519	8986.641	8501.32	1143.191	347.0786
5.324402	1288.363	10495.81	5185.933	1213.66	53683
56.67683	22.6696	9478.216	10106.32	965.3483	669.4935
56.72532	603.6156	614.7524	19275.56	139.093	58470.69
2.904726	2056.703	8992.966	6223.669	50.19381	28837.63
51.02782	3116.224	4739.587	18338.67	277.3504	
6.623705	9291.213	5387.464	5707.647		
65.86486		3767.604	18304.62		
6.187809		9566.221	10123.29		
10.50534		17132.17	14099.08		
		9669.585			
		18787.34			

Norepinephrine (pmol / µL)

Worker				Queen	
0	4-5	9-10	15	0	5
0.591201	0.169505	43.38505	125.9671	0.065755	254.438
5.562871	7.285651	8.591984	229.0202	3.519489	57.43916
1.69316	27.37127	103.3935	991.0306	4.57058	4.675058
0.123658	27.8898	146.593	76.71405	2.709221	313.0637
2.465038	2.233406	152.2876	383.5902	1.573496	6.120527
0.004971	27.99666	18.78272	2183.413	1.746952	599.7028
0.142038	19.60504	137.1103	417.852	0.990888	214.7621
0.312485	52.67638	56.78312	670.0417	2.007528	
1.244346	139.0136	200.0093	5607.511		
1.399456		143.1087	2299.572		
2.303806		306.9629	2194.764		
1.783424		607.6085	8694.622		
		380.1799			
		459.6685			

N-acetyldopamine (pmol / µL)

Worker				Queen	
0	4-5	9-10	15	0	5
6.970332	3.211837	2.30074	3.615639	38.31874	35.73728
4.320085	3.131397	18.85428	6.701898	78.99282	176.0343
6.612806	3.801563	11.85716	4.420234	5.451001	15.45371
1.589875	2.455119	8.463731	4.849207	18.65085	38.37247
11.08894	4.684387	4.703731	4.657725	21.61005	57.16249
4.021528	9.622748	2.861032	6.995913	37.18216	30.71679
0.724692	7.165978	0.479209	2.138723	37.95815	28.95292
4.003666	39.64146	0.35142	3.389894	33.45865	
1.30919	17.72461	36.80776	24.94477		
1.746866		7.447905	77.33324		
2.079603		19.85967	20.14143		
0.926543		42.85986	70.9938		
		31.94334			
		76.17156			

Tyramine (pmol / µL)

Worker				Queen	
0	4-5	9-10	15	0	5
6.128281	2.598586	37.04194	14.9207	0.992281	34.56048
5.507902	10.37546	50.44551	48.83177	2.977789	27.22992
2.340553	20.12456	58.42319	53.87759	5.464467	25.12413
1.892646	15.62932	107.2775	44.19989	6.777362	65.0167
3.306105	4.654175	57.51821	120.6059	4.082333	1.90481
0.552817	24.47239	13.83937	195.2977	1.987648	91.49468
0.189634	12.77585	67.93225	52.25112	2.647446	32.42482
0.392862	14.4795	63.85635	227.0996	0.21157	
0.084903	72.65445	25.5279	144.4436		
0.390976		70.20964	284.5568		
0.95493		81.7315	125.8595		
0.379343		133.6621	233.6234		
		63.33403			
		91.8756			

Serotonin (pmol / µL)

Worker				Queen	
0	4-5	9-10	15	0	5
0.624895	0.318732	48.26776	48.37057	1.559594	13.11714
3.119561	3.190996	10.96353	26.44091	0.44459	14.70506
0.325757	16.4108	64.65008	156.1127	1.033877	7.950344
0.362494	14.181	88.303	61.71951	1.973623	13.0512
1.405673	1.870831	83.21562	220.7992	1.287653	1.251857
0.770544	5.315006	9.206704	244.8364	0.611838	25.05964
0.991429	5.891956	103.8411	98.68984	1.085308	0.074932
0.39006	11.57492	58.17192	371.4093	0.140466	
0.349167	41.56789	42.0679	107.3741		
0.639594		41.91066	143.1505		
0.368406		73.72567	117.5058		
1.595813		135.1883	152.0953		
		66.11511			
		115.9672			
