# Supplementary material for: Caste- and age-specific venom composition of biogenic amines and the influence of diet in honey bees
Source: PLoS One. 2025 Dec 10;20(12):e0338795. doi: 10.1371/journal.pone.0338795 (PMC12694850; doi:10.1371/journal.pone.0338795)
Supplement: S2 Table — (PDF) [file pone.0338795.s002.pdf]

S2 Table. Data of concentrations of biogenic amines in the venom and maximum oocyte lengths in queenless workers.

Dopamine (pmol / μL)	Norepinephrine (pmol / μL)	*N*-acetyldopamine (pmol / μL)	Tyramine (pmol / μL)	Serotonin (pmol / μL)	Max. oocyte length (mm)
5831.7542	119.4365	6.0727	29.7507	194.8643	0.0000
6963.4547	386.4695	2.4657	29.9329	122.9492	0.0000
8155.0002	787.1989	15.7881	32.6479	151.9012	0.3214
1850.7601	36.5038	1.3213	51.1992	47.7457	0.0000
17525.2817	130.3833	4.0027	402.0015	433.8278	0.0000
14911.3119	200.6453	4.1060	292.3853	335.3226	0.0000
24194.0960	189.9996	34.7993	546.2125	618.1060	0.0000
9834.7219	1871.3118	1.0241	229.0939	420.9492	0.3784
10581.4398	530.2105	2.1740	92.4270	195.3520	0.0000
2753.3621	119.7047	4.2789	42.4331	51.7068	0.0000
9258.7853	784.7660	24.3266	180.1192	221.9009	0.0000
4229.5042	91.9444	2.8832	84.7087	65.9420	0.0000
4753.6288	367.5131	14.2902	40.7907	68.8572	0.0000
9313.0496	448.6156	18.2196	61.0712	128.6984	0.0000
8888.1655	167.4995	5.2652	165.0716	231.9279	0.0000
10324.1397	645.8744	15.0792	197.9635	238.8470	0.0000
9767.3954	240.3659	4.2433	185.9783	185.0377	0.0000
2859.3233	932.8332	16.6490	48.3919	92.9458	0.0000
4717.5014	178.2606	7.0964	57.6346	95.9787	0.0000
16411.6886	328.1771	5.8545	109.6207	150.9189	1.6071
9793.6993	310.3945	15.4899	58.4076	142.0782	0.0000
8634.9345	447.2631	4.9722	214.9365	262.9380	0.0000
11920.4471	691.6341	32.6332	129.4943	186.2782	0.0000
18871.9166	417.9351	11.4828	175.0151	406.6005	0.4731
11030.1364	364.0001	12.4248	165.4914	183.3750	0.0000
14057.2375	357.2496	16.2874	241.1260	357.7117	0.0000
12736.4854	582.7958	11.2667	104.2140	154.7030	0.0000
4339.9844	401.3812	9.7401	48.8932	128.8587	0.0000
6045.5248	118.9006	3.4280	98.8110	114.9701	0.0000
7564.3938	303.6713	9.8908	87.7982	103.7935	0.0000
5079.3770	267.0631	9.7475	78.4410	247.6240	0.0000
48243.7099	653.2162	24.8279	194.7957	260.2865	1.1786
7044.2627	903.8009	7.6234	9.3298	92.4491	0.0000
38659.3935	3199.7335	22.3858	2.3293	315.4513	0.0000
22227.5164	903.2505	27.9732	105.0940	298.5707	0.0000
49053.9154	1081.3260	32.4599	31.4759	285.4375	0.7857
7315.7032	1666.2057	12.5912	13.2293	66.8745	0.0000
39245.4919	986.2505	20.5798	12.2882	171.7562	0.0000
30063.1186	921.0487	21.3246	5.8409	164.9633	0.7143
15303.6539	2371.8168	25.1291	27.2266	70.3407	0.0000
21240.3782	569.8549	8.2231	37.3898	79.1712	0.1786
27009.7038	1938.8305	7.5961	122.9822	154.8352	0.0000
6792.6827	638.9300	3.9835	51.5171	122.1874	0.0000
25520.2285	2500.9591	18.1340	210.5899	265.4284	0.0000
12235.7269	639.6484	5.2742	94.3726	86.2852	0.0000
11816.5112	1888.4741	4.7645	62.0690	151.4194	0.0000
31918.1172	1161.4105	5.7387	118.4045	209.9242	0.3929
8953.1793	977.4395	5.7337	53.9552	84.3825	0.0000
15341.2180	799.0655	49.6676	62.2277	146.7117	0.0000
45154.7335	2157.4921	8.5349	280.1501	421.6498	0.3214
5360.7925	9717.4655	7.1055	281.7003	727.8960	0.0000
41522.8825	2345.3984	19.3971	0.4693	277.9811	0.3226
6965.4181	475.6437	2.0952	6.9601	47.5511	0.6786
25363.9898	1169.4682	13.6395	205.0974	225.3595	0.0000
10503.6842	619.8378	2.3491	75.0162	96.0996	0.3214
69042.1290	19.5106	7.2185	192.7778	290.4043	0.0000
