# Supplementary material for: Caste- and age-specific venom composition of biogenic amines and the influence of diet in honey bees
Source: PLoS One. 2025 Dec 10;20(12):e0338795. doi: 10.1371/journal.pone.0338795 (PMC12694850; doi:10.1371/journal.pone.0338795)
Supplement: S3 Table — (PDF) [file pone.0338795.s003.pdf]

S3 Table. Data of concentrations of dopamine-related substances in the hemolymph and venom in tyrosine-fed and control workers.

Tyrosine-homo (pmol / µL)

Tyr-8	Cont-8	Tyr-12	Cont-12
2227.765	1003.673	1458.241	937.3791
1701.017	91.87972	2047.16	424.1202
2514.79	1109.941	2148.523	912.7305
2814.654	1017.413	2532.971	79.17315
1912.368	1024.886	2306.624	1431.637
849.9716	1745.985	3522.304	616.9255
1761.903	511.2969	1708.593	973.9075
1346.14	729.6869	1560.297	884.9313
2763.385	900.9422	1970.134	649.7209
2743.021	850.1979	2697.719	1548.412
1473.207	672.9525	1631.86	974.1249
2356.923	505.749	1911.138	623.91
2027.796	682.3766	2056.903	1508.413
4715.691	966.6634	2278.877	1326.839
		1587.395	1005.357
		2077.523	1299.625

DOPA-hemo (pmol / µL)

Tyr-8	Cont-8	Tyr-12	Cont-12
0.542101	0.739845	0.685971	0.752617
1.218931	2.09096	0.609998	0.121067
1.859267	0.151703	1.388291	1.929778
0.150908	0.357723	4.477777	2.197539
0.031826	0.095478	1.836769	0.96986
1.287542	3.79837	1.11297	0.6641
1.324829	0.788151	1.519113	1.220695
1.932919	0.905782	2.970199	1.148796
2.661269	1.412231	2.27381	1.314321
3.326969	1.234799	11.08788	0.877004
3.632109	1.473566	1.977051	0.914691
2.376062	0.681034	2.318846	0.738342
1.748696	0.969307	1.179452	1.438049
1.254755	0.301202	1.061412	0.574556
		3.027803	1.223619
		1.104263	0.592025

Dopamine-hemo (pmol / µL)

Tyr-8	Cont-8	Tyr-12	Cont-12
0.563571	0.837345	3.410179	0.772798
0.186845	0.892539	0.738181	0.36739
0.614158	134.6475	1.740753	2.689609
0.415923	0.456743	3.514787	0.194308
0.568598	1.086872	2.511152	1.522735
0.339712	3.342064	0.642227	0.010112
1.337687	0.449639	0.746047	0.160803
0.795319	3.260114	5.733084	1.3125
0.905761	1.239517	2.055066	0.11967
2.706632	0.858181	43.46874	1.291862
2.251899	1.209649	1.709908	0.724202
0.236743	0.447902	0.665485	0.439785
0.417687	1.241717	13.10958	0.631598
3.273116	0.244373	0.628615	0.280793
		5.088999	1.585337
		3.197175	0.279359

Tyrosine-venom (pmol / µL)

Tyr-8	Cont-8	Tyr-12	Cont-12
4.700565	32.05925	11.20599	7.279426
4.508333	0.825054	4.819138	2.553619
6.381543	7.433908	3.614014	1.708649
1.79744	0.572487	1.120973	2.991383
0.021047	11.06246	4.080802	22.03035
12.86692	43.64685	6.786787	1.731098
11.21354	0.202054	2.223738	1.899469
32.1479	3.556994	6.41232	1.969156
4.196832	1.317561	13.48621	7.244965
18.57046	11.78055	6.246163	5.054487
12.45163	30.17016	9.569431	3.592574
33.97235	8.706651	8.413777	5.306068
10.21469	12.44319	3.871319	7.104994
12.45427	18.83454	8.337079	12.80311
5.188882	8.77202	7.230609	6.977253
28.49654	21.33352	7.168912	18.55492
26.366	6.553711		
17.22452	3.478593		
8.811917	13.27116		

DOPA-venom (pmol / µL)

Tyr-8	Cont-8	Tyr-12	Cont-12
1.87354	1.66724	8.970885	5.889888
1.372947	3.374898	1.080327	5.959748
1.111493	1.073601	1.486332	2.575957
1.154437	2.080261	2.048106	3.122876
2.068893	0.832357	2.057215	0.940221
0.763731	6.056375	5.004732	3.330141
1.966164	2.672636	0.093405	0.362811
0.478477	0.769204	1.67571	2.313364
0.092203	1.012975	7.769002	0.953008
1.653663	1.551059	2.144109	2.603246
2.069022	0.036096	1.429511	1.768653
2.108296	0.54073	0.785331	4.376635
2.531426	0.992281	3.186797	0.855433
1.546812	0.937076	0.38735	0.926483
3.394069	0.743149	1.4513	2.173582
0.39148	2.060703	0.821656	1.253026
1.410307	0.018048		
4.024193	4.194771		
1.664935	2.798916		

Dopamine-venom (pmol / µL)

Tyr-8	Cont-8	Tyr-12	Cont-12
1513.769	2589.623	7155.701	1765.516
7112.877	3945.687	1332.256	2699.366
1823.784	1411.677	11000.26	2238.217
4027.921	3630.234	7136.554	3305.247
3019.409	2160.355	10935.25	2633.328
5420.399	1090.181	8452.26	6836.964
2351.786	1874.782	8149.483	2155.874
2999.985	2955.879	14330.31	4945.633
5659.875	2945.392	3420.927	3679.927
2488.105	1218.09	9326.547	5758.119
1274.037	1233.991	11114.48	7754.318
1593.103	959.6696	7640.319	5067.641
1483.462	3359.269	8189.74	4194.544
3401.751	1242.361	6616.261	3669.748
2937.258	1210.071	6708.886	3746.595
1352.981	4597.418	9277.935	3910.481
1734.933	1851.786		
2667.655	2173.175		
2226.259	1416.229		
