# Supplementary material for: Caste- and age-specific venom composition of biogenic amines and the influence of diet in honey bees
Source: PLoS One. 2025 Dec 10;20(12):e0338795. doi: 10.1371/journal.pone.0338795 (PMC12694850; doi:10.1371/journal.pone.0338795)
Supplement: S4 Table — (PDF) [file pone.0338795.s004.pdf]

S4 Table. Data of concentrations of dopamine-related substances in the hemolymph and venom in royal jelly-fed and control workers.

Tyrosine-hemo (pmol / μL)

| Roy-8 | Cont-8 | Roy-12 | Cont-12 |
|---|---|---|---|
| 3425.448 | 387.1146 | 2594.606 | 803.6267 |
| 1300.897 | 331.2647 | 4035.426 | 905.428 |
| 3276.12 | 504.1085 | 3567.67 | 683.8441 |
| 3519.054 | 346.786 | 2845.879 | 1153.446 |
| 2879.988 | 359.8602 | 3223.049 | 1818.529 |
| 3321.977 | 417.6824 | 3113.764 | 839.5899 |
| 3918.328 | 236.9585 | 2861.652 | 607.9341 |
| 2522.18 | 514.4696 | 2697.636 | 862.5646 |
| 3216.329 | 1004.081 | 3893.648 | 585.6036 |
| 3784.443 | 404.2304 | | |

DOPA-hemo (pmol / μL)

| Roy-8 | Cont-8 | Roy-12 | Cont-12 |
|---|---|---|---|
| 9.873945 | 0.934355 | 2.206323 | 0.616493 |
| 1.618336 | 0.01063 | 1.892049 | 0.006279 |
| 0.000559 | 0.004196 | 2.769166 | 1.882631 |
| 4.854447 | 0.07693 | 2.532087 | 0.711708 |
| 7.941454 | 0.558095 | 8.602088 | 1.431327 |
| 26.65372 | 0.465499 | 3.721563 | 0.520431 |
| 9.454884 | 0.010351 | 2.784109 | 1.456473 |
| 3.193031 | 2.140623 | 0.700972 | 0.123186 |
| 2.905765 | 0.023364 | 4.257556 | 4.424695 |
| 3.854086 | 0.018871 | | |

Dopamine-hemo (pmol / μL)

| Roy-8 | Cont-8 | Roy-12 | Cont-12 |
|---|---|---|---|
| 2.79975 | 0.823708 | 2.768618 | 2.028941 |
| 4.291837 | 0.961384 | 2.139686 | 1.253556 |
| 72.32602 | 0.006525 | 5.75774 | 2.42371 |
| 1.449511 | 0.036158 | 4.160455 | 0.811381 |
| 4.47102 | 0.371096 | 3.414279 | 0.459685 |
| 1.691382 | 0.792685 | 8.29459 | 0.9875 |
| 3.087727 | 0.01487 | 5.934749 | 4.407736 |
| 2.188816 | 1.456786 | 11.76638 | 0.333194 |
| 0.842102 | 0.017214 | 4.506736 | 0.536901 |
| 1.768373 | 0.434991 | | |

Tyrosine-venom (pmol / μL)

| Roy-8 | Cont-8 | Roy-12 | Cont-12 |
|---|---|---|---|
| 15.2746 | 1.750058 | 6.387962 | 4.960644 |
| 0.685248 | 7.16298 | 0.715732 | 0.100249 |
| 0.064242 | 2.6018 | 0.115342 | 0.056444 |
| 8.951048 | 1.830896 | 2.274384 | 0.885922 |
| 0.374745 | 0.697025 | 0.06761 | 0.813649 |
| 3.620035 | 0.134908 | 0.707183 | 0.023314 |
| 2.754374 | 0.149898 | 0.171785 | 0.755856 |
| 0.114326 | 0.085745 | 5.771443 | 0.06217 |
| 89.39157 | 0.064023 | 0.02106 | 1.347844 |
| | | 0.002354 | 0.051783 |

DOPA-venom (pmol / μL)

| Roy-8 | Cont-8 | Roy-12 | Cont-12 |
|---|---|---|---|
| 0.044305 | 0.315674 | 1.581001 | 1.760938 |
| 0.134498 | 0.237349 | 0.303447 | 0.52042 |
| 0.023735 | 1.024557 | 0.266458 | 0.632217 |
| 0.067249 | 0.221526 | 3.144533 | 0.711014 |
| 1.107629 | 0.083072 | 0.053457 | 0.853741 |
| 5.621217 | 1.063324 | 1.533833 | 0.251563 |
| 0.062304 | 0.353057 | 6.813706 | 0.453475 |
| 1.891334 | 0.111255 | 1.528592 | 1.373113 |
| 8.36894 | 0.04493 | 0.015765 | 0.025224 |
| | | 0.627256 | 0.031715 |

Dopamine-venom (pmol / μL)

| Roy-8 | Cont-8 | Roy-12 | Cont-12 |
|---|---|---|---|
| 1409.883 | 896.9713 | 4229.468 | 6.824316 |
| 2984.61 | 845.652 | 1941.378 | 2849.65 |
| 4566.243 | 265.0604 | 4480.376 | 1594.007 |
| 7094.293 | 905.0069 | 1742.839 | 1169.076 |
| 2043.982 | 1102.062 | 4684.498 | 2434.282 |
| 3204.022 | 479.2137 | 3525.517 | 680.763 |
| 1548.721 | 633.5098 | 4277.78 | 733.8239 |
| 2101.033 | 2303.389 | 2525.26 | 1733.876 |
| 4286.916 | 278.1599 | 2286.797 | 981.9958 |
| | | 5598.466 | 827.2286 |
