# Supplementary material for: Caste- and age-specific venom composition of biogenic amines and the influence of diet in honey bees
Source: PLoS One. 2025 Dec 10;20(12):e0338795. doi: 10.1371/journal.pone.0338795 (PMC12694850; doi:10.1371/journal.pone.0338795)
Supplement: S5 Table — (PDF) [file pone.0338795.s005.pdf]

S5 Table. P values of the Steel-Dwass multiple comparison test between different ages in workers (Figs. 1A-E).

	0 vs. 5 days	0 vs. 10 days	0 vs. 15 days	5 vs. 10 days	5 vs. 15 days	10 vs. 15 days
Dopamine	0.0191	0.0000	0.0000	0.0098	0.0068	0.8383
Norepinephrine	0.0104	0.0000	0.0000	0.0146	0.0000	0.0129
N-acetyldopamine	0.2941	0.1134	0.0634	0.8970	0.9363	0.9997
Tyramine	0.0010	0.0000	0.0000	0.0063	0.0033	0.5297
Serotonin	0.0143	0.0000	0.0000	0.0007	0.0000	0.0517
